# Cardiogenic Airflow in the Lung Revealed Using Synchrotron-Based Dynamic Lung Imaging

**DOI:** 10.1038/s41598-018-23193-w

**Published:** 2018-03-21

**Authors:** Stephen Dubsky, Jordan Thurgood, Andreas Fouras, Bruce R. Thompson, Gregory J. Sheard

**Affiliations:** 1 Monash University, Department of Mechanical & Aerospace Engineering, Melbourne, 3800 Australia; 2 Biomedical Imaging Research Institute, Department of Biomedical Sciences, Cedars-Sinai Medical Center, Los Angeles, California, 90048 USA; 3 4Dx Limited, Melbourne, 3004 Australia; 4 Monash University, Department of Medicine, Melbourne, 3800 Australia; 5 The Alfred Hospital, Allergy Immunology & Respiratory Medicine, Melbourne, 3004 Australia

## Abstract

The beating heart is known to produce pressure and airflow oscillations in the lungs of mammals. This phenomenon is often disregarded as detailed measurement of its effects in the lung have hitherto not been possible. Previous studies have attempted to measure the effect of these oscillations on gas mixing. However, the results have proven inconclusive, due to the lack of a direct measurement tool capable of flow measurement throughout the entire bronchial tree. Here we present the first detailed measurement of cardiogenic oscillations, using synchrotron-based dynamic lung imaging of live mechanically ventilated mice. The results demonstrate large flow oscillations and pendelluft in the airways due to the mechanical action of the beating heart. Using a virtual tracer modelling analysis we show that cardiogenic oscillations produced up to 4 times increased gas mixing, but only in the absence of tidal ventilation. The results highlight the importance of considering this often-disregarded phenomenon when investigating lung function, particularly in situations where tidal ventilation is reduced or absent.

## Introduction

The effective exchange of oxygen and carbon-dioxide between air and blood within the lungs is arguably the most important process for sustaining mammalian life. In order to transport oxygen from fresh air to the gas exchange surfaces, and to remove carbon dioxide from within the lungs to the atmosphere, the incoming gas must be mixed with the resident gas that remains within the lungs. Understanding the mechanisms behind this transport is critical for understanding the effects of lung disease on lung function, for designing optimum mechanical ventilation (MV) techniques, and for optimizing inhaled drug delivery.

Airflow in the lungs is typically thought of as being generated by the diaphragm and chest wall, yet pressure oscillations caused by the beating heart, termed cardiogenic oscillations (CO), can also generate significant airflows^1^. These oscillations can be observed from birth^2^, and have been discussed as a potential mechanism for apneic respiration (i.e. respiration during breath-holding) in crocodilians^3^ and hibernating mammals^4,5^. Cardiogenic oscillations have also been shown to influence aerosol deposition in humans^6–8^.

Cardiogenic oscillating flows were first measured directly in the lobar airways by West and Hugh-Jones^1^, who postulated that the phase shifts between these oscillations may cause internal flows with the potential to enhance gas mixing in the lungs.

Hyperpolarized He^3^ MRI has been used to investigate cardiogenic flows in the lungs of humans^9,10^. These studies found significant cardiogenic flows in the lungs that resulted in flow of air between the left and right lungs^9^ and also between the upper and lower regions of the left lung^10^. Due to the spatial resolution of the MRI technique used, these investigations were limited to measuring flow only at the main bronchi. In fact, Collier *et al*.^10^ concluded that quantitative measurement of cardiogenic flow at the lobar scale or finer is necessary to fully assess the magnitude and consequence of cardiogenic effects in the lungs.

Recently, we have developed functional lung imaging using synchrotron-based 4D-CT, and have demonstrated the capability to measure airflow throughout the entire airway tree in mice^11^. This method has been applied to models of disease, revealing the regional patterns of disease^12^. Additionally, a two-dimensional variant of this method has revealed patterns of lung tissue oscillation under high-frequency ventilation^13^ and forced oscillation technique for lung function measurement^14^.

Using these advances, we present here, for the first-time, detailed measurement of tissue displacement, tissue expansion and flow in the airway tree due to cardiogenic oscillations within the mouse lung. These quantitative measurements allowed us to digitally isolate the effects of mechanical ventilation and cardiogenic oscillations, without the need to alter normal physiological conditions. These measurements allowed us to establish the pattern and magnitude of cardiogenic flows within the lung. Using a numerical modelling analysis based on virtual tracers, we use this new data to investigate the contribution of cardiogenic oscillations to the enhancement of gas mixing.

To investigate the relative contributions of each type of flow (MV and CO), analysis was performed on each of three mice under 4 states: (1) DIFF: zero flow (molecular diffusion only), (2) CARD: cardiogenic oscillations only (no mechanical ventilation) (3) VENT: mechanical ventilation (no cardiogenic oscillations) (4) BOTH: cardiogenic oscillations plus mechanical ventilation. The magnitude and distribution of the resulting tissue displacement, expansion and airway flow due to MV and CO was investigated. The contribution of MV and CO to gas mixing for each state was deduced through a novel gas tracer technique, whereby massless tracers allow visualization and analysis of the gas transport within the lung (see Methods for details).

## Results

### Magnitude and distribution of cardiogenic oscillations

The dynamic tissue motion due to mechanical ventilation and cardiogenic oscillations are visualized in Supplementary Movie S1. The influence of the heart on the lung tissue is clearly apparent throughout both the left and right lungs.

**Figure 1:**
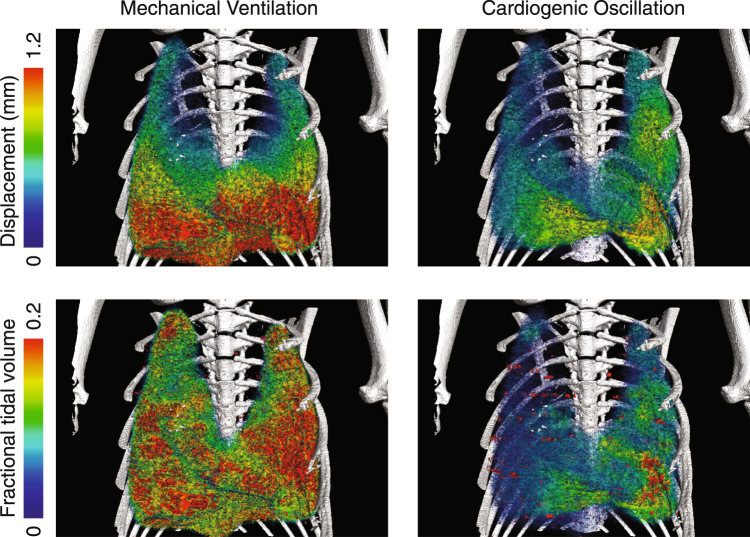
Regional tissue displacement (top) and tidal volume (bottom) for mechanical ventilation (left) and cardiogenic oscillations (right) for Mouse 2. The displacement and tidal volume generated by the cardiogenic oscillations of the heart are localized to the vicinity of the heart.

The local displacement and tidal volume of lung tissue resulting from MV and CO for Mouse 2 are shown in Fig. 1. Displacement was calculated as the total translation of each region of tissue over the relevant cycle (either mechanical ventilation cycle or heart beat). The fractional tidal volume is defined as the change in volume of each region of tissue over the cycle (maximum volume minus minimum volume) divided by the initial volume of the region. MV caused large displacements of the lung tissue, with a distinct cranio-caudal gradient. The tidal volume under MV is more evenly distributed throughout the lung. The CO causes significant displacement and expansions of the lung tissue, of similar maximum magnitude to the MV in some areas adjacent to the heart. The effects of CO on the lung tissue appear highly localized to the vicinity of the heart (predominantly in the left lobe and cardiac lobe).

**Figure 2:**
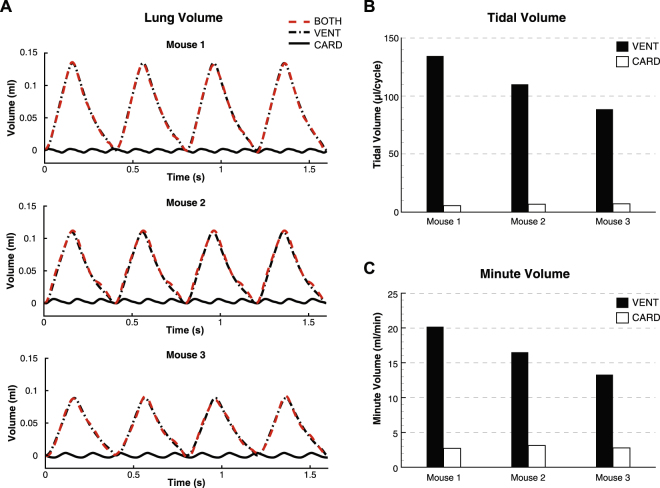
Lung volume (**A**), tidal volume (**B**) and minute volume (**C**) generated by mechanical ventilation and cardiogenic oscillations. The oscillations in global lung volume measured at the trachea are smaller in amplitude than the tidal ventilation, but occur at a higher frequency.

Total lung volume vs. time for CARD, VENT and BOTH are shown in Fig. 2A. The BOTH and VENT curves are very similar, although some differences are apparent due to the addition of CO. The relative magnitude of CARD is clearly lower than for VENT and BOTH, but the frequency of the cycle is increased, reflecting the frequency of the heartrate relative to the ventilation cycle. The faster cycle of the heart (~3× the ventilation cycle) leads to a relative increase in minute volume (compared to tidal volume), as is apparent in Fig. 2B,C, resulting in minute volumes generated by CO in isolation to be between 13% and 20% of the minute volume generated by MV.

### Flow in the airways

**Figure 3:**
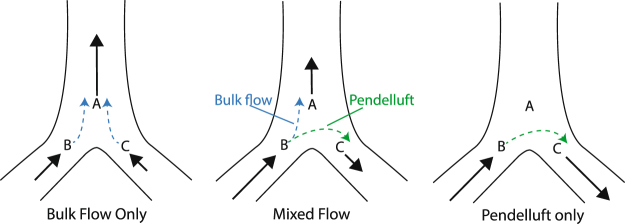
Flow regimes at a bifurcation in the airway tree. Bulk flow transports gas between the trachea and alveoli, while pendelluft acts to redistribute gas between different lung regions.

Gas flow through any bifurcation in the airway tree can be classified into one of three regimes (Fig. 3). Bulk flow is the unidirectional flow from parent to siblings (or vice versa) that transports gas between the trachea to the alveoli. Pendelluft is flow between two sibling airways that transports gas internally between adjoining lung regions, which is thought to occur during the transition between inspiration and expiration due to differences in compliance between lung units^15^. A combination of these two flows may also be present (mixed flow).

A novel gas tracer modelling analysis was developed to visualize the gas transport within the lungs due to MV and CO. The airway tree was numerically seeded with virtual massless tracers (representing gas molecules) that follow the measured flow through the airways (see Methods for details). The tracer paths were visualised to reveal the transport of gas within the airway tree.

**Figure 4:**
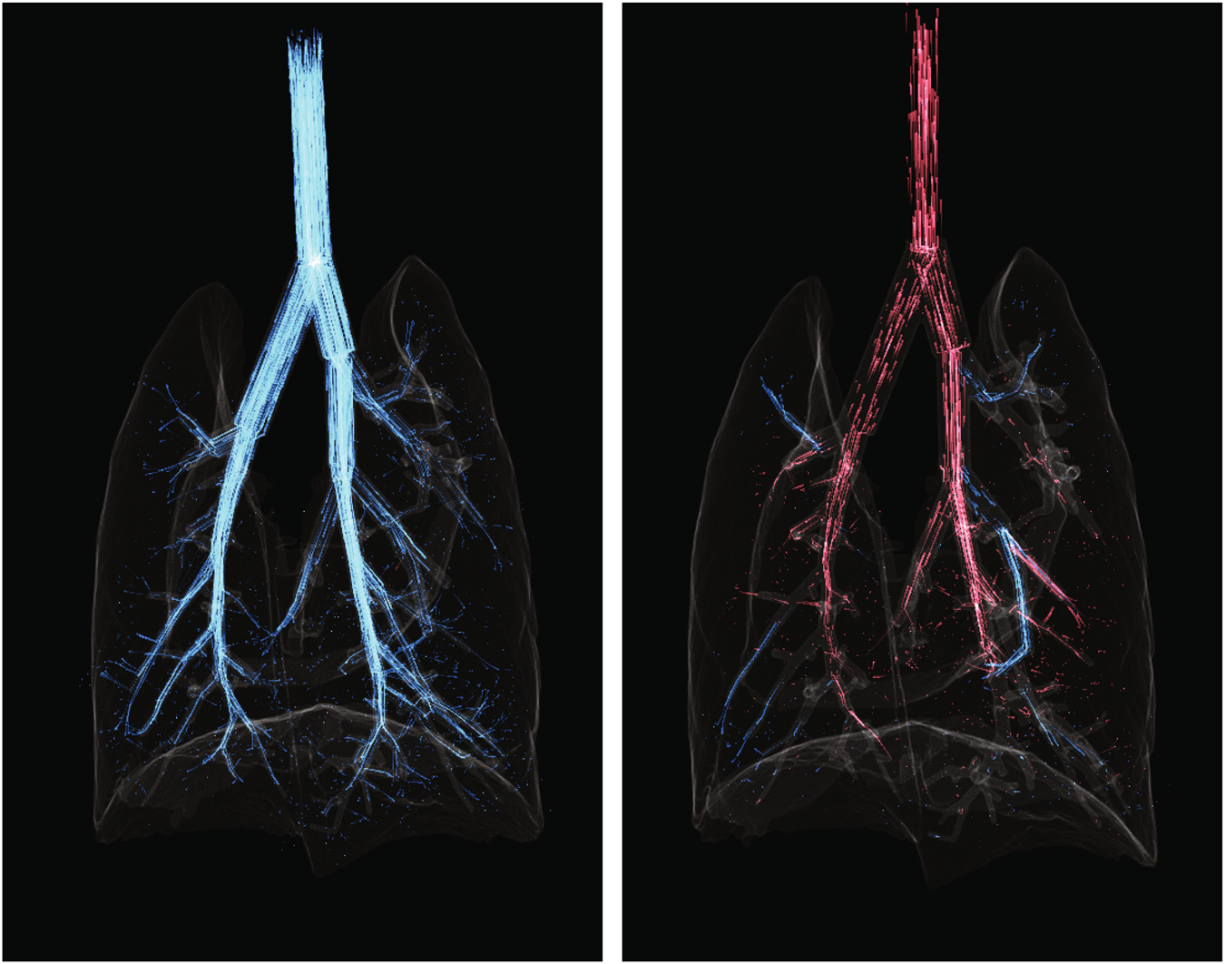
Single frames from Supplementary Movie S2 (left) and Supplementary Movie S3 (right), showing tracer paths in mechanical ventilation during inspiration (left) and cardiogenic flows (right). Red paths indicate flow travelling in the direction of ascending generations (ie from trachea to the periphery) and blue indicates flow travelling through descending generations. The ventilation flows demonstrate a single serial path of tracers from the trachea to the periphery. In contrast, the cardiogenic flows show pendelluft and redistribution of flows between lung regions, with both blue and red tracer paths apparent at this time-point.

The dynamic tracer visualizations for MV and CO are shown in Fig. 4 and Supplementary Movies 2 and 3. During MV inspiration, the gas flows are predominantly direct from the trachea to the periphery. During expiration, gas flows are predominantly from the periphery to the trachea via a similar path but reversed pathway as MV inspiration. However, during CO, the gas oscillates along these pathways, and also exhibits pendelluft flow during much of the cycle.

The change in total lung volume, as shown in Fig. 2A, may underestimate the effects of CO, as any redistribution of gas internally to the lung due to pendelluft will not incur a change in total volume. To more accurately assess the contribution of CO to gas transport in the lungs, we defined Total Internal Flow: Q_tot_ = |Q_A_| + |Q_B_| + |Q_C_|, where Q_A_, Q_B_ and Q_C_ denote the flow through airways A, B and C respectively, as labeled in Fig. 3. The bulk flow can be determined as Q_bulk_ = |Q_A_ + Q_B_ + Q_C_| and pendelluft as the difference Q_pend_ = Q_tot_ − Q_bulk_. Note that although Fig. 3 shows a single bifurcation, these calculations can be performed across the entire airway tree.

**Figure 5:**
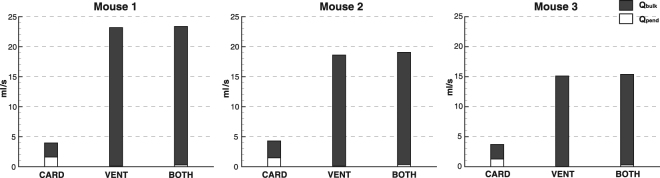
Pendelluft and bulk flow for CARD, VENT and BOTH. Q_tot_ is the sum of Q_pend_ and Q_bulk_, represented as the total height of the columns in the figure. Although Q_tot_ is far greater in VENT and BOTH, Q_pend_ is greater for CARD.

Figure 5 shows Q_pend_, Q_bulk_, and Q_tot_ for CARD, VENT, and BOTH for each mouse, integrated over time and expressed as ml/s. Q_tot_ is significantly higher for VENT and BOTH when compared to CARD. However, a large proportion of Q_tot_ for CARD is made up of pendelluft. (35–41%). In fact, Q_pend_ for CARD is between 14 and 21 times larger than for VENT, and around 5 times larger than for BOTH.

### Gas mixing

**Figure 6:**
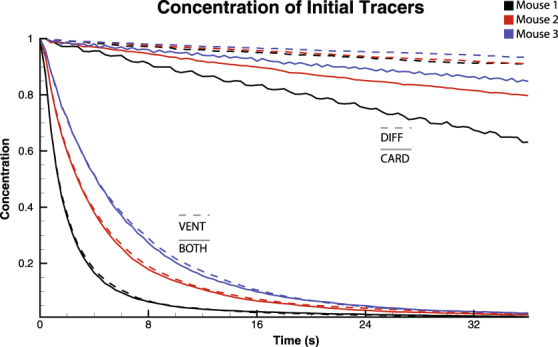
Concentration profiles for gas mixing. Four cases are shown: DIFF, CARD, VENT, and BOTH. Cardiogenic flows produce a significant increase in gas mixing over the diffusion only case. However, the addition of cardiogenic flows provides only a minor enhancement to mixing under mechanical ventilation.

The contribution of cardiogenic oscillations to gas mixing was investigated using the gas tracer analysis. The lung was initially seeded with particles (Type A), representing resident gas. Tracers incoming to the trachea were assigned to Type B, representing fresh gas. As the incoming tracers mixed with the initial tracers, and the initial tracers are expelled through the trachea opening, the concentration of Type A tracers within the lung is reduced. This is analogous to an inert gas washout. Enhanced gas mixing within the lung will increase the proportion of resident gas within the expired air, thus increasing the rate at which the Type A particle concentration is reduced within the lung. Figure 6 shows concentration through time of Type A particles, calculated at end inspiration, for each mouse under the 4 states: DIFF, CARD, VENT and BOTH. Compared to DIFF, the introduction of CO in the CARD state increased mixing in the lung considerably. However, with MV present, the addition of CO provided only a small enhancement to the gas mixing within the lung.

**Figure 7:**
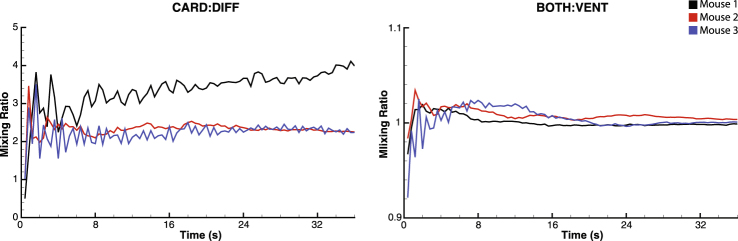
Mixing ratio CARD:DIFF (left) and BOTH:VENT (right). Cardiogenic oscillations produce up to 4 times mixing compared to diffusion only. However, in the presence of mechanical ventilation, mixing enhancement due to cardiogenic oscillations is approximately 2%. Note the vertical axis scales are different for the two plots for clarity.

The mixing enhancement due to CO is shown in Fig. 7. This enhancement was calculated as the ratio of the concentrations of fresh gas in the system (Type B tracers) with cardiogenic oscillations compared to without (CARD:DIFF and BOTH:VENT) over time. CO provided up to 4 times greater mixing in the absence of mechanical ventilation (Fig. 7, left). However, only minimal enhancement is seen with MV present (Fig. 7, right).

## Discussion

Using high-speed dynamic synchrotron-based imaging we have, for the first time, acquired detailed measurements of the effects of cardiogenic oscillations on lung tissue displacement, expansion and flow in the lungs. Additionally, we used a novel gas tracer modelling analysis to investigate the effects of cardiogenic oscillations on gas mixing. Tissue displacement and tidal volume maps showed large perturbations due to CO that were most prominent near the heart. Qualitative and quantitative analysis of the airflow within the airway tree showed significant pendelluft due to CO, but this was reduced in the presence of mechanical ventilation. Likewise, CO produced enhanced mixing during the breath-hold state (up to 4 times), but only limited enhancement was seen due to CO in the presence of MV (~2%). This suggests that pendelluft present within the lung may be an effective gas mixing mechanism, albeit only in the presence of reduced tidal flows.

The effect of cardiogenic oscillations on gas mixing have been previously studied experimentally using gas washout or bolus dispersion measurements^16–21^. The use of gas washout or bolus dispersion can provide an overall inference of the gas mixing enhancement due to cardiogenic effects. However, these techniques are limited to either global lung measurements, or at best measurements in only a small number of airways. These studies produced conflicting results on the significance of cardiogenic mixing. For example, Engel *et al*.^16^ found up to 5 times increase in mixing due to cardiogenic oscillations, whilst Schell *et al*.^20^ concluded that there was a limited effect of cardiac action on gas mixing.

A potential contributing factor to these conflicting results is the varied experimental conditions used that may have significantly affected the resulting measurements, such as using open-chest post-mortem measurements^16–18^, temporary cardiac arrest to stop the heart^20,21^, or exercise to alter the cardiac output^19^. Additionally, these studies have performed measurements either under breath-hold conditions^16,18,21^ or in the presence of tidal breathing^19,20^.

Importantly, our study has shown the increase in mixing when adding CO in apnea was much larger when compared to its addition to MV. The flow patterns shown in Supplementary Movies S2 & S3 and Fig. 4 demonstrate the mechanism behind this. During apnea, CO can produce significant pendelluft. This is not possible during MV as the bulk flow cannot be overcome by the CO to drive the flow between the sibling branches during most of the ventilation cycle. This results in a weaker mixing effect in the presence of MV. This may explain inconsistencies in the literature regarding the contribution of CO to enhanced gas mixing, which used measurements acquired either in the presence or absence of breathing.

The results were consistent with previous direct flow measurements and imaging studies on humans and dogs^1,9,10^, conducted under breath-hold conditions, which showed out-of-phase cardiogenic flows that result in pendelluft. Our results showed that, in the mouse, internal pendelluft flows contribute up to 41% of the total flow due to CO during breath-holding. Measurements of the flow or pressure at the mouth will therefore greatly underestimate the effects of CO and cannot be used for assessing the magnitude of internal cardiogenic flows.

Although the measurements taken in this study were performed on mice, the conclusions are likely to be relevant for humans. Collier *et al*. measured tidal volumes due to CO in humans of around 53 mL, or approximately 9% of total lung capacity (~6 L). Similarly, our measurements showed tidal volumes of between 5.5% and 7.1% of total lung capacity in the mouse (~1 mL). Therefore, the relative magnitude of the cardiogenic flows is comparable between humans and mice, and the relative effects may also be comparable. A notable difference is that Reynolds number and Womersley number in humans is much greater than in mice. This can lead to turbulent flows in the trachea and main bronchii, and a highly skewed velocity profile with significant secondary flows at the bifurcations. This increases gas mixing at these locations. A more comprehensive flow model that removes the parabolic flow profile assumption would be required to apply the tracer modelling analysis in human lungs to fully quantify the effects of CO in humans.

Our results show that in the absence of tidal ventilation, the cardiogenic oscillations may assist in respiration and mixing within the lung. There are situations in which this may be relevant for humans. For example, humans exhibit respiratory pauses during normal activities, such as swallowing and talking. Long breath-holds are often performed during free diving, swimming, and inhaled drug delivery. Apneic events during sleep are common, with an estimated range of 9% to 38% of the population exhibiting more than 5 apneic events (>10 s) per hour during sleep^22^ and respiratory pauses of greater than 3 s are common during sleep in normal infants^23^.

It should also be noted that normal breathing in humans typically includes an expiratory pause of around 2 s. These pauses were not present in the mechanical ventilation in this study. CO may therefore provide greater enhancement of mixing in normal breathing in humans than was apparent in the mice in this study.

Although the results showed greater gas mixing due to CO in the absence of tidal flows, this mixing was still much less than that generated by the mechanical ventilation. As such, the mixing caused by cardiogenic oscillations may not be sufficient to significantly affect gas exchange. Further modelling that includes gas exchange across the air/blood barrier in the terminal compartments is necessary to investigate the physiological significance of the mixing induced by cardiogenic oscillations.

In conclusion, this study has presented the first detailed measurement of cardiogenic oscillations across the whole lung, revealing the influence that cardiogenic oscillations have on ventilation in the mammalian respiratory system. The effects of cardiogenic oscillations on gas flow and mixing were shown to be greatest during periods where the tidal ventilation is absent, as this allows the existence of pendelluft. The results highlight the importance of considering this often-disregarded phenomenon when investigating lung function, particularly in conditions where tidal ventilation is reduced or absent.

## Methods

Synchrotron imaging projection data from baseline scans of control mice acquired as part of a previous experiment^24^ were used for analysis. The animal procedures and imaging used are detailed in that study, and briefly outlined here for convenience.

### Animal Procedure

All animal procedures were approved by the SPring-8 Animal Care Committee and Monash University’s School of Biomedical Science’s Animal Ethics Committee, and all methods were performed in accordance with the relevant guidelines and regulations. All studies were conducted in experimental hutch 3 of beamline 20B2 in the Biomedical Imaging Centre at the SPring-8 synchrotron in Japan.

Adult male Balb/C mice were anaesthetised using sodium pentobarbitone (i.p.; 70 mg/kg) and tracheostomised, Anaesthesia was maintained throughout the experiment with top-up of sodium pentobarbitone every 30 minutes (i.p.; 30 mg/kg). Positive pressure ventilation was delivered through a custom designed ventilator (based on that described in Kitchen *et al*.^25^ with 120 ms inspiration time, 280 ms expiration time, 10 cmH_2_O inflation pressure and 2 cmH_2_O positive end expiratory pressure (PEEP), consistent with the recommendations of Glaab *et al*.^26^: PEEP is required to maintain functional residual capacity, as active inspiratory muscle tone is reduced in anesthetized mice^26^. Electrocardiography (ECG) was attached to record the precise timing of both the ventilation cycle and the cardiac cycle, and airway pressure was measured at the entrance to the tracheal tube. Each mouse was ventilated for at least 5 minutes prior to imaging to allow it to stabilize after anaesthetization and surgery.

### Imaging

Imaging was conducted using a modification of the dynamic computed tomography method described in Dubsky *et al*.^11^. Briefly, phase-contrast images were acquired at the SPring-8 synchrotron, Japan, at the BL20B2 beam-line. Images were acquired at 50 fps using a PCO.edge sCMOS detector (PCO AG, Germany), optically coupled with a scintillator crystal. During imaging, the animal was placed upright in a custom-built holder, which was mounted on a 5-axis motor controller to provide stable rotation during the 3 minute scan. The custom designed ventilator provided stable, pressure-controlled ventilation, and provided triggering to the imaging system for synchronization with the ventilation cycle. Single-image phase retrieval^27^ and simultaneous algebraic reconstruction technique^28^ was used for CT reconstruction. Imaging parameters resulted in high-resolution CT with an isotropic voxel size of 15 μm.

### Double-gated CT reconstruction

A retrospective double-gating procedure was developed to separately bin projection images into 19 ventilation phases and into 8 cardiac phases. The images were binned into 19 ventilation phases based on the image acquisition triggered by the mechanical ventilation. Separately, the images were binned into 8 cardiac phases based on the ECG trace. Motion blur due to ventilation on the cardiac phase CTs was reduced by only utilizing images acquired at an airway pressure of <4 cmH_2_O (as measured by the ventilator), thus effectively producing cardiac phase data at approximately expiratory pressure.

Subsequent CT reconstruction resulted in two 4DCT movies, one showing lung motion due to ventilation, and the other showing lung motion due to the beating heart (see Supplementary Movie S1). This data isolates the effects of ventilation and heart action without requiring physical intervention (ie. by pausing ventilation or stopping the heartbeat), which would disrupt the physiological processes occurring within the system.

### Airway tree segmentation

The airway tree was segmented according to the process described in Dubsky *et al*.^24^. This process utilizes the vesselness filter described by Frangi *et al*.^29^. This Hessian-based filter uses analysis of the eigenvalues of the image intensity Hessian-matrix at different spatial scales (*σ*) in order to assign a probability value to each voxel. This value describes the probability of a cylinder (in our case an airway) being present at each voxel. The spatial scale that yields the maximum vessel probability at any point may be used to estimate the vessel diameter. The 4DCT images were processed using the vesselness filter to yield a vessel probability field. This was segmented using a flood-fill, providing a binary image of the airway tree. Auto-skeletonisation (Avizo, FEI software, USA) was then used to find the centerline of the airways. The scale of the vesselness filter that yielded the highest vessel probability at each centerline point of the airway tree was used as an estimate of the diameter of the airway at that point. These estimates were averaged across each airway segment to yield the average diameter of each airway segment. This method provides robust, unsupervised diameter estimation across the entire airway tree.

### Tissue expansion and airway flow measurement

Tissue displacement, expansion and airway flow was calculated using the cross-correlation velocimetry procedure described previously^11,12^. Briefly, three-dimensional cross-correlation was performed between two successive interrogation regions, with the maximum correlation value representing the modal displacement of the lung tissue within that region. The cross-correlation analysis was performed with interrogation regions of 32 × 32 × 32 voxels (representing a 480 μm^3^ region) with a regular spacing of 16 voxels between the centres of adjacent interrogation regions. The expansion field of lung tissue was calculated from the local gradients in the lung displacement.

The segmented airway tree was associated with the lung expansion maps to calculate the time-varying airflow throughout the bronchial tree. The method utilises the local ventilation values of lung tissue to directly infer the local airflow through each airway supplying a given region of tissue. The supplying airways are defined as the terminal airways of the airway segmentation, that is the airways with a parent but no daughter branches. Each interrogation region is associated with its closest supplying airway. The expansion of the regions supplied by an airway (occurring due to gas flowing into/out of that region) equals the flow through that airway. Assuming negligible compressibility effects, the principle of continuity dictates that at each bifurcation, the flow through a parent segment must equal the sum of the flow through its daughter segments. The flow through the entire tree can therefore be calculated by recursively summing the airflows in daughter segments at each bifurcation to calculate the airflow through the parent segment.

### Gas tracers and mixing calculation

A numerical gas tracer method was developed to visualize the internal flows and deduce the effects of flow on gas mixing within the lungs. To investigate the relative contributions of each type of flow (diaphragmatic flow, cardiogenic flow), the tracer analysis was performed on each data set under 4 conditions: (1) DIFF: zero flow (molecular diffusion only), (2) CARD: cardiogenic oscillations, (3) VENT: mechanical ventilation (4) BOTH: cardiogenic oscillations + mechanical ventilation. The concentration of initial (Type A) particles in the system was plotted against time for each condition. The method consisted of the following steps:

#### Geometry simplification and peripheral airway modelling

To increase efficiency and reduce the effects of segmentation errors, the airway geometries were first simplified into straight cylinders with a single radius. This allows each airway to be completely specified by its two end-points and its radius. The segmentations defined airways down to approximately 85–100 µm, corresponding to generation 14 in the Oldham & Robinson model^30^. To model the peripheral airways and the acini, each segmented distal airway was replaced with a trumpet model, according to the geometry specified by Oldham and Robinson. An acinar compartment was placed at the end of each trumpet with equivalent cross-sectional area and volume as generation 23 in the Oldham & Robinson model.

#### Tracer advection

The geometry was numerically seeded with massless tracers. Each airway and terminal compartment is initially seeded with a number of tracers proportional to its volume, so that the tracer density throughout the domain is constant. Each tracer is defined by its containing airway, its location within that airway in cylindrical co-ordinates. The initial tracers were designated as Type A (representing the resident gas) and new tracers entering via the trachea opening were designated Type B (representing fresh gas from the atmosphere).

During each time-step, tracers were advected according to the gas velocity at its location, using a 4^th^ order Runge-Kutta time integration. The image analysis produces a single bulk-flow for each airway over time. A Poiseuille (parabolic) velocity profile is assumed for all airways at each time-point. As the Womersley number in the system is <1 throughout the system, the effects of oscillations on the velocity profile are negligible. Linear interpolation in time was used to oversample the flow data obtained from the imaging.

As tracers pass through a bifurcation, to preserve mass continuity they are randomly assigned to one of the other two airways based on the ratio of flow between the two candidate airways. The tracer maintained its relative radius location in its new containing airway.

The volume flux into the trachea was integrated over time, and at each timestep the integer value of this integral (multiplied by the initial volumetric tracer concentration) was used to insert tracers (Type B) at the trachea opening at a randomly assigned cross-sectional position, with the remaining decimal value being conserved in the integrated volume flux for subsequent time-steps. Tracers that traverse out of the trachea at the proximal boundary (i.e. leave through the trachea opening due to flow out of the system, typically during expiration) are removed from the simulation.

Tracers that enter the terminal compartments from the terminal airways were added to a tally of tracers within that compartment, and no longer subjected to advection or diffusion updates. The volume flux from the terminal compartments into the terminal ‘trumpet’ airways was monitored, and tracers introduced into the terminal airways from the terminal compartments according to the accumulated magnitude of this flux. The type of tracer that was introduced was randomly assigned in proportion to the concentration of Type A and Type B tracers contained in the compartment.

#### Diffusion

Molecular diffusion was modelled using the mathematical technique known as random walks. At every time-step, each tracer is randomly displaced according to a normal distribution with zero mean and standard deviation of *6Dt*, where *D* is the diffusion coefficient, and *t* is the timestep, and in a random direction in 3D. The diffusion coefficient was chosen was 0.23 cm^2^/s which is the self-diffusivity coefficient of nitrogen at body temperature^31^. This was chosen in order to model the intrinsic mixing of air within the lungs, which is predominantly nitrogen. This displacement is sub-stepped by 100 times. If a tracer crosses an airway wall during a sub-step, the direction is randomly reset to simulate interaction with a solid boundary. If the displacement enters a bifurcation, the tracer is randomly assigned to one of the three airways of the bifurcation according to the ratio of cross-sectional areas, thus maintaining mass continuity. Tracers that cross the system boundary (ie. pass out of the trachea entrance or into a terminal compartment) are dealt with as in the advection step.

### Data availability

The datasets generated during and/or analysed during the current study are available from the corresponding author on reasonable request.

## Electronic supplementary material

Supplementary Information

Supplementary Movie S1

Supplementary Movie S2

Supplementary Movie S3

## References

[1] West JB, Hugh-Jones P (1961). Pulsatile gas flow in bronchi caused by the heart beat. J. Appl. Physiol..

[2] Hathorn MKS (2000). Cardiac Contraction Affects Respiratory Airflow in the Term Newborn Infant. Pediatr Res.

[3] Farmer CG (2010). The Provenance of Alveolar and Parabronchial Lungs: Insights from Paleoecology and the Discovery of Cardiogenic, Unidirectional Airflow in the American Alligator (*Alligator mississippiensis*). Physiol. Biochem. Zool..

[4] Szewczak JM, Jackson DC (1992). Apneic oxygen uptake in the torpid bat, Eptesicus fuscus. J. Exp. Biol..

[5] Sullivan SG, Szewczak JM (1998). Apneic oxygen uptake in the torpid pocket mouse Perognathus parvus. Physiol. Zool..

[6] Prisk GK, Sá RC, Darquenne C (2013). Cardiogenic mixing increases aerosol deposition in the human lung in the absence of gravity. Acta Astronaut..

[7] Darquenne C, Paiva M, Prisk GK (2000). Effect of gravity on aerosol dispersion and deposition in the human lung after periods of breath holding. J. Appl. Physiol..

[8] Scheuch G, Stahlhofen W (1991). Effect of Heart Rate on Aerosol Recovery and Dispersion in Human Conducting Airways After Periods of Breathholding. Exp. Lung Res..

[9] Sun Y, Butler JP, Ferrigno M, Albert MS, Loring SH (2013). “Ventilatory alternans”: A left–right alternation of inspiratory airflow in humans. Respir. Physiol. Neurobiol..

[10] Collier GJ (2015). Observation of cardiogenic flow oscillations in healthy subjects with hyperpolarized ^3^He MRI. J. Appl. Physiol..

[11] Dubsky S, Hooper SB, Siu KKW, Fouras A (2012). Synchrotron-based dynamic computed tomography of tissue motion for regional lung function measurement. J. R. Soc. Interface.

[12] Stahr, C. S. *et al*. Quantification of heterogeneity in lung disease with image-based pulmonary function testing. *Sci. Rep*. **6** (2016). 10.1038/srep29438 PMC4962033 27461961

[13] Thurgood J (2012). Functional Lung Imaging during HFV in Preterm Rabbits. PLoS ONE.

[14] Thurgood J (2016). Imaging lung tissue oscillations using high-speed X-ray velocimetry. J. Synchrotron Radiat..

[15] Greenblatt EE, Butler JP, Venegas JG, Winkler T (2014). Pendelluft in the bronchial tree. J. Appl. Physiol..

[16] Engel LA (1973). Gas mixing during breath holding studied by intrapulmonary gas sampling. J. Appl. Physiol..

[17] Engel LA, Wood LD, Utz G, Macklem PT (1973). Gas mixing during inspiration. J. Appl. Physiol..

[18] Fukuchi Y, Roussos CS, Macklem PT, Engel LA (1976). Convection, diffusion and cardiogenic mixing of inspired gas in the lung; an experimental approach. Respir. Physiol..

[19] Drechsler DM, Ultman JS (1984). Cardiogenic mixing in the pulmonary conducting airways of man?. Respir. Physiol..

[20] Schell, J. M. *et al*. Significance of Cardiogenic Mixing in Dog Lungs. In *Oxygen Transport to Tissue XII* 609–614 (Springer, 1990). 10.1007/978-1-4684-8181-5_69 2096663

[21] Cybulsky IJ (1987). Contribution of cardiogenic oscillations to gas exchange in constant-flow ventilation. J. Appl. Physiol..

[22] Senaratna CV (2017). Prevalence of obstructive sleep apnea in the general population: A systematic review. Sleep Med. Rev..

[23] Ellingson R, Peters J, Nelson B (1982). Respiratory pauses and apnea during daytime sleep in normal infants during the first year of life: longitudinal observations. Electroencephalogr. Clin. Neurophysiol..

[24] Dubsky S (2017). Assessment of airway response distribution and paradoxical airway dilation in mice during methacholine challenge. J. Appl. Physiol..

[25] Kitchen MJ (2010). A new design for high stability pressure-controlled ventilation for small animal lung imaging. J. Instrum..

[26] Glaab, T., Taube, C., Braun, A. & Mitzner, W. Invasive and noninvasive methods for studying pulmonary function in mice. *Respir. Res*. **8** (2007). 10.1186/1465-9921-8-63 PMC2039738 17868442

[27] Paganin D, Mayo SC, Gureyev TE, Miller PR, Wilkins SW (2002). Simultaneous phase and amplitude extraction from a single defocused image of a homogeneous object. J. Microsc..

[28] Andersen AH, Kak AC (1984). Simultaneous algebraic reconstruction technique (SART): a superior implementation of the ART algorithm. Ultrason. Imaging.

[29] Frangi, A. F., Niessen, W. J., Vincken, K. L. & Viergever, M. A. Multiscale vessel enhancement filtering. in *International Conference on Medical Image Computing and Computer-Assisted Intervention* 130–137 (Springer, 1998).

[30] Oldham MJ, Robinson RJ (2007). Predicted Tracheobronchial and Pulmonary Deposition in a Murine Asthma Model. Anat. Rec. Adv. Integr. Anat. Evol. Biol..

[31] Winn EB (1950). The temperature dependence of the self-diffusion coefficients of argon, neon, nitrogen, oxygen, carbon dioxide, and methane. Phys. Rev..

