# Supplementary material for: Cardiogenic Airflow in the Lung Revealed Using Synchrotron-Based Dynamic Lung Imaging
Source: Sci Rep. 2018 Mar 21;8:4930. doi: 10.1038/s41598-018-23193-w (PMC5862895; doi:10.1038/s41598-018-23193-w)
Supplement: Supplementary file 1 — Supplementary Information [file 41598_2018_23193_MOESM1_ESM.pdf]

Cardiogenic Airflow in the Lung Revealed Using Synchrotron-Based Dynamic Lung Imaging

Supplementary Information

Stephen Dubsky[1,*], Jordan Thurgood[1], Andreas Fouras[1,2], Bruce Thompson[3,4], and Gregory J Sheard[1]

[1]Monash University, Department of Mechanical & Aerospace Engineering, Melbourne, 3800, Australia
[2]4Dx Limited, Melbourne, 3004, Australia
[3]Monash University, Department of Medicine, Melbourne, 3800, Australia
[4]The Alfred Hospital, Allergy Immunology & Respiratory Medicine, Melbourne, 3004, Australia

*Dr Stephen Dubsky
Department of Mechanical & Aerospace Engineering
Monash University, VIC 3800, Australia
Email: stephen.dubsky@monash.edu

Description of Supplementary Movies

Supplementary Movie S1:

Four-dimensional Computed Tomography of the lungs during mechanical ventilation and cardiogenic oscillations. Time slowed down by a factor of ~2.

Supplementary Movie S2:

Tracer paths during mechanical ventilation. Blue paths indicate flow travelling in the direction of descending generation (ie from trachea to the periphery) and red indicates flow travelling through ascending generations. The ventilation flows demonstrate a single serial path of tracers from the trachea to the periphery. Time stamp notes number of time-steps (0.5ms per time-step), and time is slowed down by a factor of ~60.

Supplementary Movie S3:

Tracer paths during cardiogenic oscillations. Blue paths indicate flow travelling in the direction of descending generation (ie from trachea to the periphery) and red indicates flow travelling through ascending generations. The cardiogenic flows show pendelluft and redistribution of flows between lung regions, with both blue and red tracer paths apparent at the same time, at various times throughout the heart cycle. Time stamp notes number of time-steps (0.5ms per time-step), and time is slowed down by a factor of ~60.
